# *ZNF350* promoter methylation accelerates colon cancer cell migration

**DOI:** 10.18632/oncotarget.26353

**Published:** 2018-12-04

**Authors:** Hiroki Tanaka, Yuki Kuwano, Tatsuya Nishikawa, Kazuhito Rokutan, Kensei Nishida

**Affiliations:** ^1^ Department of Pathophysiology, Institute of Biomedical Sciences, Tokushima University Graduate School, Tokushima 770-8503, Japan

**Keywords:** DNA methylation, EMT, cell migration, *ZNF350*, colon cancer cells

## Abstract

Diversification of transcriptomic and epigenomic states may occur during the expansion of colorectal cancers. Certain cancer cells lose their epithelial characters and gain mesenchymal properties, known as epithelial-mesenchymal transition (EMT), and they aggressively migrate into the non-tumorigenic extracellular matrix. In this study, we isolated a subpopulation with accelerated baseline motility (MG cells) and an immotile one (non-MG cells) from a colon cancer cell line (HCT116). Gene expression signatures of the MG cells indicated that this subpopulation was likely an EMT hybrid. The MG cells substantially lost their migratory properties after treatment with a methyltransferase inhibitor, 5-azacytidine, suggesting a role of DNA methylation in this process. Global transcriptome assays of both types of cells with or without 5-azacytidine treatment identified 640 genes, whose expression might be methylation-dependently down-regulated in the MG cells. Global methylation analysis revealed that 35 out of the 640 genes were hyper-methylated in the MG cells. Among them, we focused on the anti-oncogene ZNF350, which encodes a zinc-finger and BRCA1-interacting protein. Notably, ZNF350 knockdown accelerated migration of the non-MG cells, while overexpression of ZNF350 in the MG cells significantly impaired their migration. Finally, pyrosequence analysis together with dual luciferase assays of serially truncated fragments of the ZNF350 promoter (-268 to +49 bp) indicated that three hyper-methylated sites were possibly responsible for the basal promoter activity of ZNF350. Taken together, our results suggest that hyper-methylation of the ZNF350 proximal promoter may be one of the crucial determinants for acquiring increased migratory capabilities in colon cancer cells.

## INTRODUCTION

Malignant transformation of cancer cells represents the acquisition of specific capabilities including uncontrolled proliferation, resistance to apoptosis, increased migration, and aggressive invasion. These phenotypic alterations imply changes in numerous cell signaling pathways and are mainly acquired after alterations of the cell genome. Tumor cells can adapt to changes in microenvironments, resulting in having heterogeneous subpopulations. As a consequence, certain tumor cells lose their epithelial characters and gain mesenchymal properties, described as epithelial-mesenchymal transition (EMT) [1, 2], and they aggressively migrate into the non-tumorigenic extracellular matrix. Cell migration plays a pivotal role during invasion and metastasis in various types of tumors [1]. Recent studies suggest that epigenetic mechanisms, such as changes in DNA methylation status, are involved in this transition [3].

The DNA methyltransferase catalyzes the addition of a methyl group on to the cytosine residue in the 5′ position of a cytosine-guanine (CG) dinucleotide, leading to the formation of 5-methylcytosine [4]. These CG dinucleotides, also known as CpGs, are distributed in islands, particularly concentrated in the promoter regions of various genes. In humans, about 50–70% of genes have a promoter rich in CpG islands [5]. The methylation of CpG islands plays an important role in gene regulation and usually results in transcriptional repression. Moreover, there are at least three major CpG methylation-dependent mechanisms that are involved in oncogenesis. The first is hypo-methylation of the cancer genome, resulting in the activation of putative oncogenes and genome instability [6]. The second is focal hyper-methylation in the promoters of tumor suppressor genes, thus inactivating their transcription. This mechanism is highly prevalent and well-illustrated by the hyper-methylation of the promoters of *RB1* in retinoblastoma [7], of the *MLH1* promoter in colon cancer [8, 9], and of the *BRCA1* promoter in breast cancer [10]. The third mechanism is direct mutagenesis. Methylated CpG sites are hotspots for C to T transition mutations. Furthermore, the methylation of CpG islands facilitates the binding of chemical carcinogens and increases the risk of UV-induced mutations [11]. Although the function and downstream effects of CpG methylation are widely accepted, the role of this process in heterogeneous subpopulations of cells with regards to the increased migratory properties of certain cells is largely unknown.

In this study, we purified a subpopulation of cells from the colon cancer cell line HCT116, which had high migration capacity. Separation and purification of these cells were performed using a transwell apparatus, a classical chemotactic assay initially described by Boyden [12]. Gene expression signatures indicated that this subpopulation was an EMT hybrid. We employed global DNA methylation and pyrosequence analyses, and found that this hybrid possessed hyper-methylated CpG sites in the proximal promoter of *ZNF350* encoding zinc finger protein 350 (ZNF350/ZBRK1). We show here that hyper-methylation of the *ZNF350* promoter may be one of the crucial determinants for acquiring increased migratory capabilities in colon cancer cells.

## RESULTS

### Selection and characterization of a subset of HCT116 cells with a highly motile phenotype

**Figure 1:**
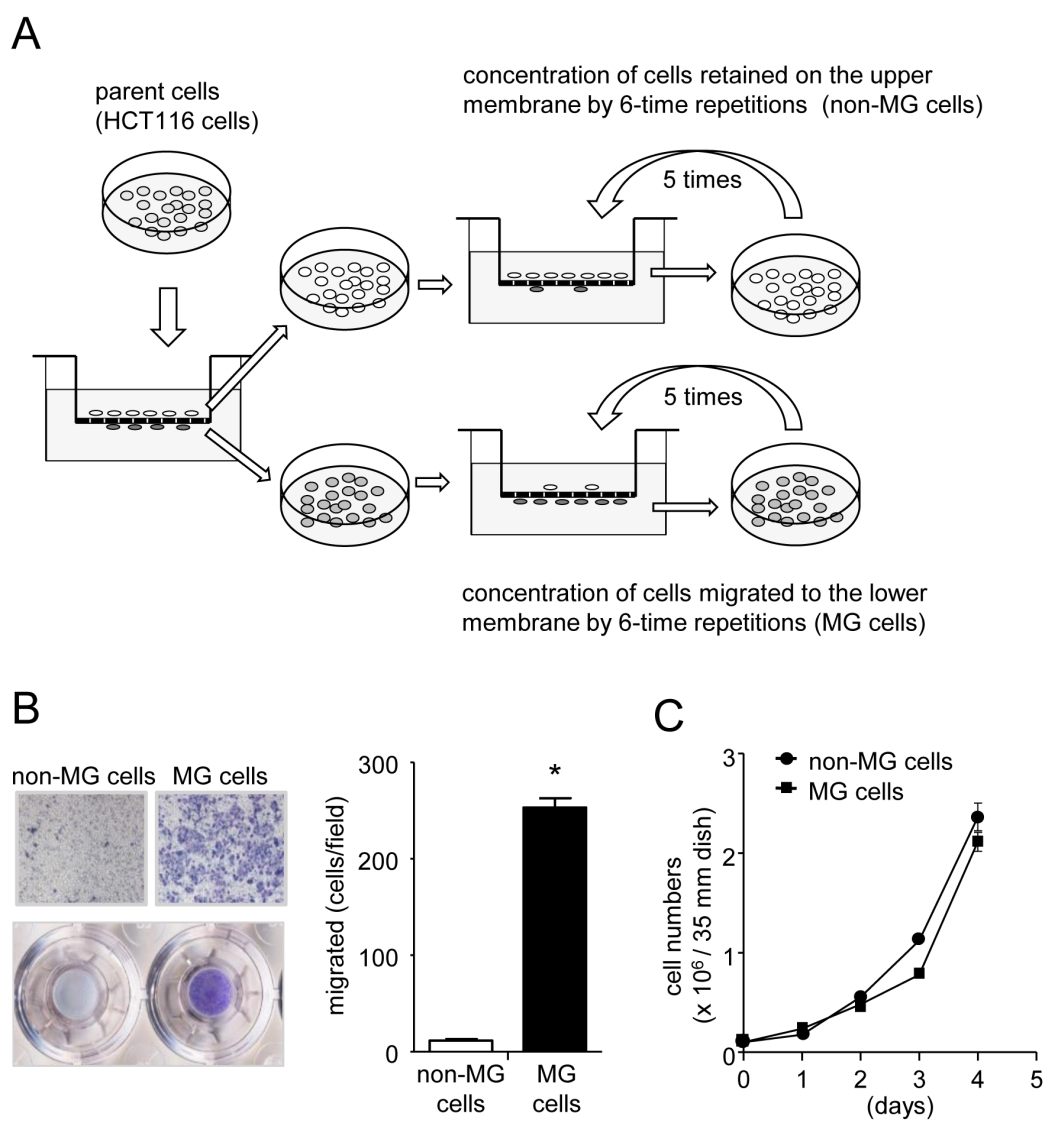
Preparation of highly motile and immotile subpopulations of HCT116 cells **(A)** Schematic representation of the methods used for the isolation and enrichment of the highly motile and immotile cell populations. HCT116 cells were seeded in serum-free media on the upper membrane of a Boyden chamber and allowed to migrate towards media containing 10% of FBS in the lower chamber. After incubation for 48 h, cells remaining on the upper membrane (non-MG cells) or cells migrating to the lower side of the membrane (MG cells) were collected. Both types of cells were separately cultured in 10% FBS-containing media. The cells were enriched by repeating the same procedure five times. **(B)** Purified MG cells or non-MG cells were seeded in serum-free media on the upper membrane of a Boyden chamber and allowed to migrate towards media containing 10% of FBS in the lower chamber. After incubation for 24 h, migrating cells were subjected to Diff-Quick staining. The numbers of migrating cells were counted. Values represent the means ± SD (n = 4). ^*^P < 0.01, unpaired Student’s *t*-test. **(C)** The growth rates of the MG and non-MG cells in the 10% FBS-containing medium for the indicated times. Data are presented as the means ± SD (n = 4).

To investigate the role of DNA methylation in the acquisition of enhanced migratory capabilities in colon cancer cells, we isolated two subpopulations of HCT116 cells, one that had accelerated baseline motility and another that was largely immotile, using the transwell migration assay system (Figure 1A). After cell enrichment with repetitive migration assays, the cells that migrated (MG cells) exhibited a distinctly higher migratory capacity than the cells that did not migrate (non-MG cells) (Figure 1B). There was no difference in their growth rate (Figure 1C), indicating that the difference in migration of the cell subpopulations was independent of their mitogen activity.

### Gene expression signatures in MG cells

**Figure 2:**
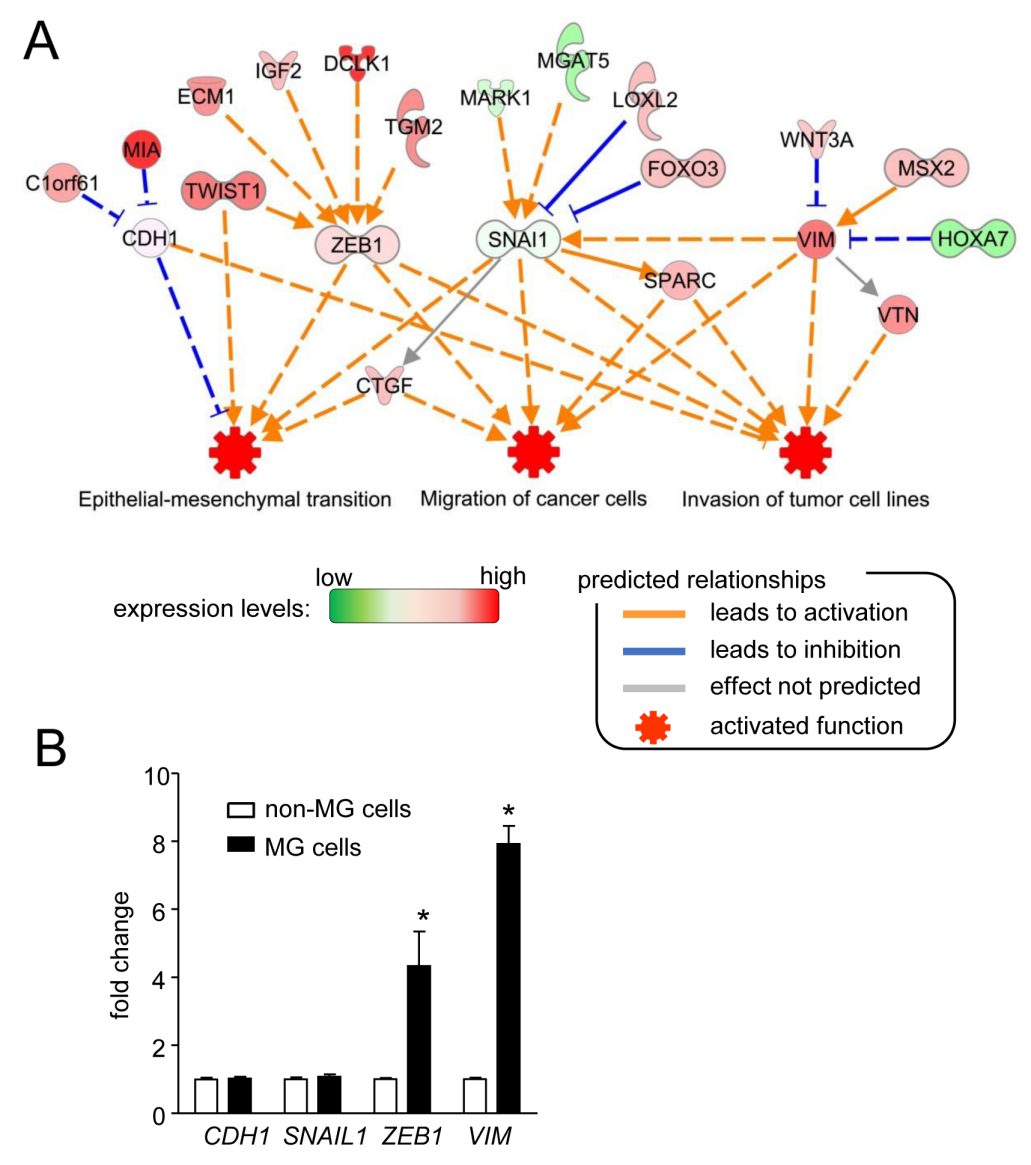
Gene expression signatures of the MG cells **(A)** Ingenuity pathway analysis (IPA) of functional networks related to epithelial-mesenchymal transition (EMT), migration of cancer cells, and invasion of tumor cell lines, focusing on four major EMT marker genes (*CDH1*, *ZEB1*, *SNAIL1*, and *VIM*). Up-regulated and down-regulated genes in the MG cells are shown in red and green, respectively. **(B)** *CDH1*, *SNAIL1*, *ZEB1,* and *VIM* mRNA levels in the MG and non-MG cells were assayed by qPCR. mRNA expression in the MG cells was calculated with the comparative ΔΔCt method using *GAPDH* mRNA as an endogenous quantitative control and are expressed as the relative changes compared to their expression in control non-MG cells. Data are presented as the means ± SD (n = 4). ^*^P < 0.05, unpaired Student’s *t*-test.

To determine the mechanism underlying the observed increase in migration of the MG cells compared with that of the non-MG cells, we analyzed their gene expression profiles using a whole human genome microarray (Agilent Technologies, Santa Clara, CA, USA). We found that 4,178 genes in total were differentially expressed; the MG cells up-regulated 1,800 genes and down-regulated 2,378 genes, compared to the non-MG cells. An Ingenuity Pathway Analysis (IPA; Qiagen, Hilden, Germany) of the 4,178 differentially expressed genes ranked significantly affected molecular and cellular functions as follows: 1) Cell Death and Survival (283 molecules, *P* = 2.88E-03 – 2.18E-05), 2) Cellular Function and Maintenance (179 molecules, *P* = 2.70E-03 – 2.97E-05), 3) Molecular Transport (170 molecules, *P* = 2.79E03 – 5.82E-05), 4) Cellular Movement (213 molecules, *P* = 2.08E-03 – 6.05E-05), and 5) Cellular Compromise (41 molecules, *P* = 2.08E-03 – 6.05E-05). At the same time, we focused on the expression of four genes encoding E-cadherin (*CDH1*), vimentin (*VIM*), zinc finger E-box binding homeobox 1 (*ZEB1*), and SNAIL protein (*SNAIL*), which are known to encode protein markers that play a crucial role in migration-related functions [1]. Using IPA, we selected 17 genes connected to the four genes (*CDH1*, *VIM*, *ZEB1*, and *SNAIL*) from 213 differentially expressed, cellular movement-related genes and we built a functional network as shown in Figure 2A. Figure 2A also shows functional connections of the 17 and 4 (*CDH1*, *VIM*, *ZEB1*, and *SNAIL*) genes to EMT, Migration of Cancer Cells, and Invasion of Tumor Cell Lines. As shown in Figure 2A, *ZEB1* mRNA expression was up-regulated in association with up-regulation of many of its activator genes (e.g., *TWIST1*, *ECM1*, *IGF2*, *DCLK1*, and *TGM2*) in the MG cells. *VIM* expression was also increased along with its associated activator gene (*MSX2*), while its inhibitor gene (*HOXA7*) was down-regulated. In contrast, *CDH1* expression was unchanged in spite of the up-regulated expression of *C1ORF61* and *MIA*, two inhibitory factors of this gene. For *SNAIL1*, its activator genes (*MAPK1* and *AGAT5*) were down-regulated, while its inhibitors (*LOXL2* and *FOXO3*) were up-regulated. However, there was no change in the expression of this gene in the MG cells. Notably, the mRNA expression changes of these four marker genes were confirmed by qPCR (Figure 2B); the MG cells significantly increased *ZEB1* and *VIM* mRNA levels, while *CDH1* and *SNAIL* mRNA levels were not changed in the cells, compared with those in the non-MG cells. These data suggested that the MG cell subpopulation was composed of EMT intermediates. This phenotype also appeared to be stable, as the migratory capabilities and the EMT marker gene expression of the MG and non-MG cells stayed the same even after five passages under standard cell culture conditions (Supplementary Figure 1).

### Comparison of DNA methylation profiles between the MG and non-MG cells

**Figure 3:**
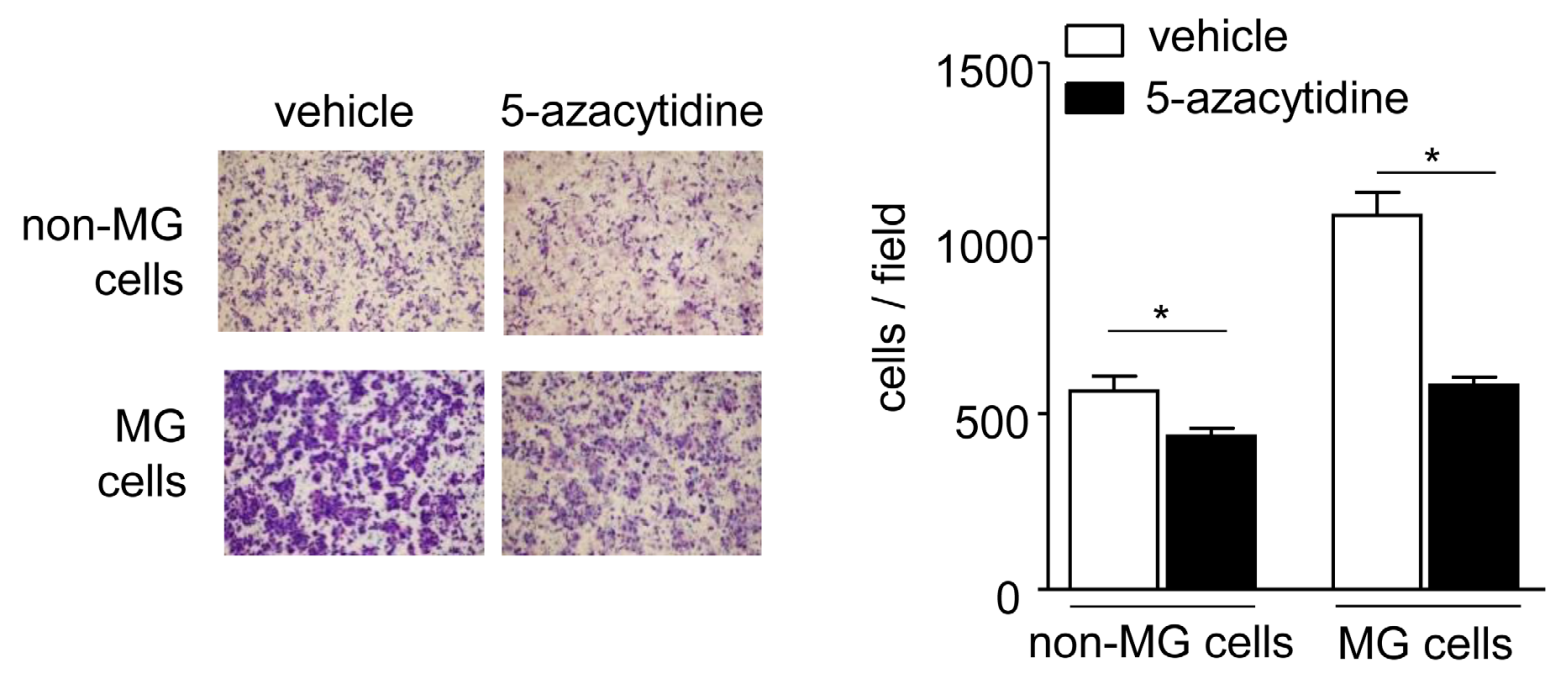
Effects of 5-azacytidine treatment on cell migration The MG and non-MG cells were treated with 5 μM 5-azacytidine for 48 h in 5% FBS-containing medium, and then they were seeded in 5% FBS-containing medium on the upper side of a transwell chamber and allowed to migrate towards 10% FBS-containing medium in the lower chamber. After incubation for 24 h, migrating cells were subjected to Diff-Quick staining (left panels) and counted (right panel). Data are presented as the means ± SD (n = 4). ^*^P < 0.05, unpaired Student’s *t*-test.

To evaluate the possible role of DNA methylation in the observed changes in gene expression and the subsequently increased migration in the MG cells, we first examined the effects of a methyltransferase inhibitor, 5-azacytidine, on cell migration, and found that treatment with 5-azacytidine significantly reduced the migration of both MG and non-MG cells (Figure 3). Particularly, the MG cells completely lost their property of accelerated migration.

**Figure 4:**
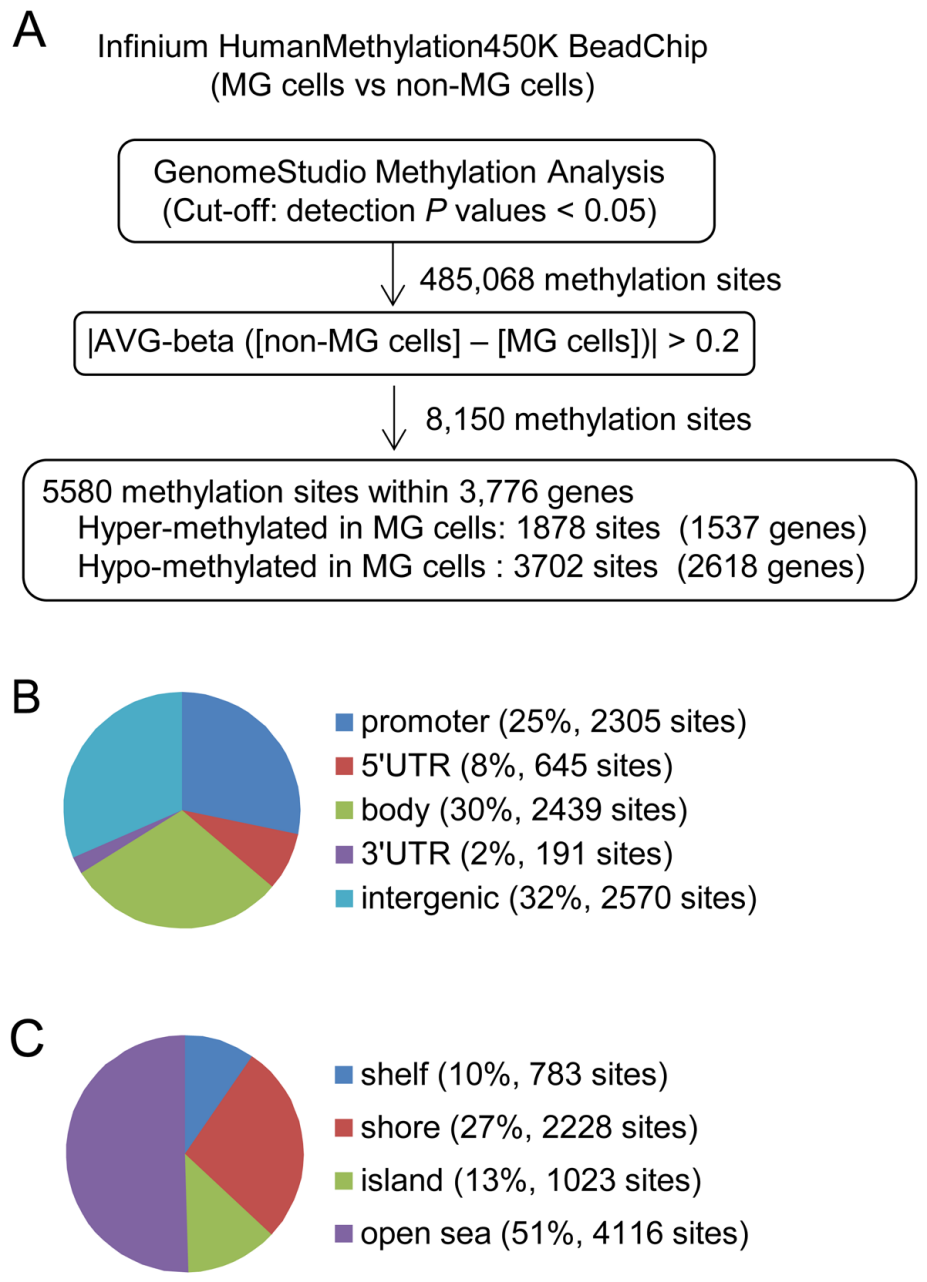
Analysis of the differentially methylated CpG sites between MG and non-MG cells **(A)** Flow chart of the processes used to identify differentially methylated CpG sites between the MG and non-MG cells. **(B)** Pie chart shows the distribution of the 5,580 differentially methylated CpG sites in the following functional genomic groups: promoter, 5’UTR, body, 3’UTR, and intergenic. Percentages and CpG counts are indicated in parentheses. **(C)** Pie chart shows the distribution of the 5,580 differentially methylated CpG sites over CpG islands, CpG shores, CpG shelves, and open sea. Percentages and CpG counts are indicated in parentheses.

Based on these results, we then compared global methylation profiles between the MG and non-MG cells using the Infinium HumanMethylation450 BeadChip platform (450K; Illumina Inc., San Diego, CA, USA). The DNA methylation levels of the CpG sites on the array were analyzed using the GenomeStudio Methylation Module v1.9.0 (Illumina) with a *P* < 0.05 cut-off threshold. Of the 485,068 sites with significant signals, 8,150 sites were differentially methylated in the MG cells compared to the non-MG cells (Figure 4A). These data were assessed using β-values that represent the percentage of methylation, which ranged from 0 (completely unmethylated) to 1 (fully methylated). This was also conducted using the GenomeStudio methylation software after color balance adjustment and background corrections in the same chip. Among the 8,150 differentially methylated CpG sites in the MG cells, 5,580 sites were distributed in 3,776 annotated genes (1,878 CpG sites in 1,537 genes were hyper-methylated, while 3,702 sites in 2,618 genes were hypo-methylated) (Figure 4A). The genomic distribution of the 8,150 differentially methylated CpG sites regarding their respective location to genes and CpG context are shown in Figure 4B and Figure 4C, respectively. Twenty-five percent of the differentially methylated sites were located in the promoter regions, and 13% and 27% of them were distributed in the CpG islands and shores, respectively.

### Methylation-dependent down-regulation of gene expression in MG cells

**Figure 5:**
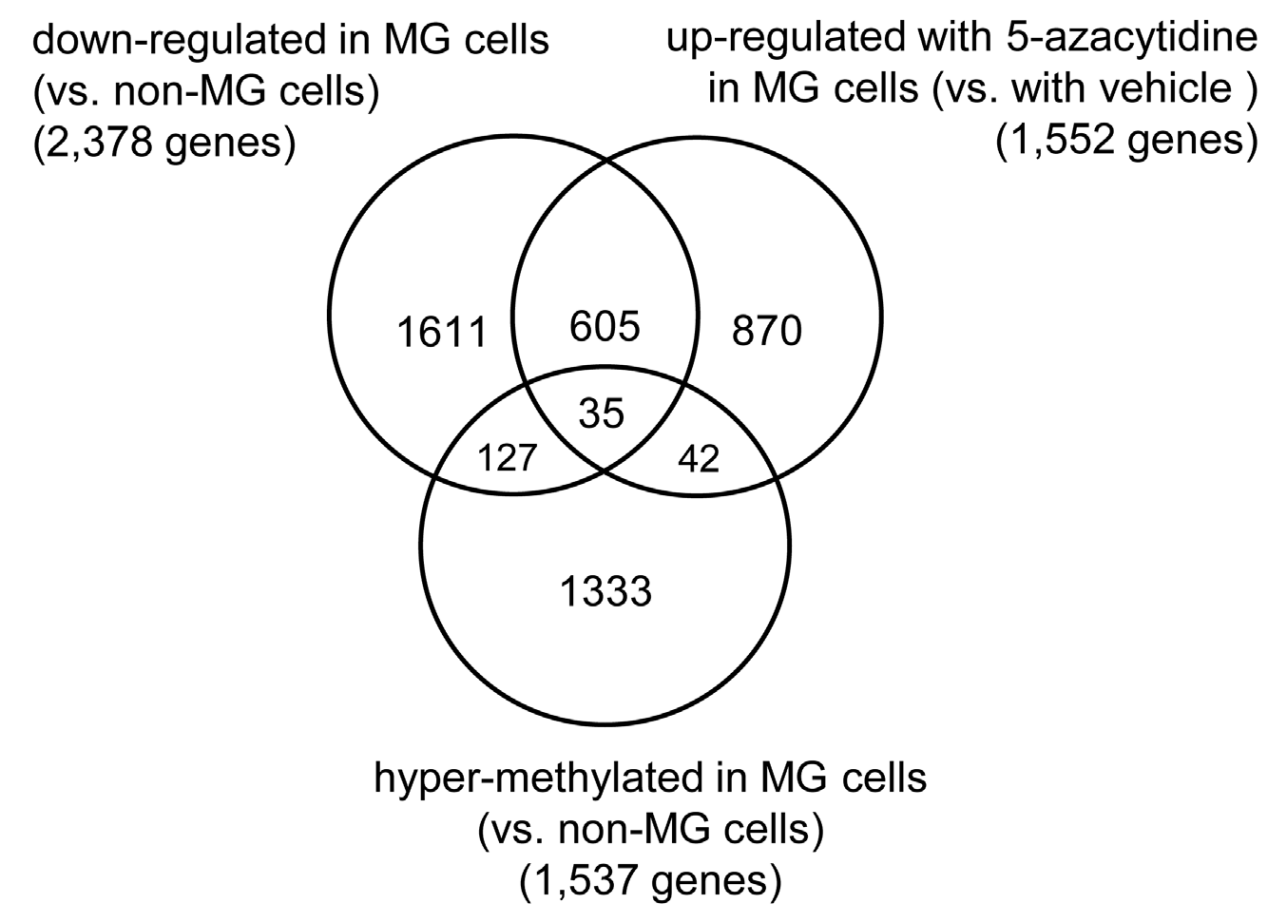
Identification of hyper-methylated and down-regulated genes in the MG cells Using a human mRNA microarray, we identified 640 genes whose expression was down-regulated in the MG cells compared to the non-MG cells and was significantly recovered after treatment of the MG cells with 5-azacytidine. Global DNA methylation was also evaluated between the MG and non-MG cells, and hyper-methylated CpG sites in 1,537 genes were identified. The Venn diagram shows that 35 out of the 640 genes are hyper-methylated in the MG cells.

Our data indicate that the expression of 2,378 genes was significantly down-regulated in the MG cells compared to that in the non-MG cells, while treatment with 5-azacytidine up-regulated the expression of 1,552 genes in the MG cells. A Venn diagram of these gene sets suggests that DNA methylation may play a role in the down-regulation of 640 genes in the MG cells (Figure 5). Among these, a global methylation analysis indicated that 35 genes were hyper-methylated in the MG cells (Table 1). In addition, we performed Pearson’s correlation coefficient analysis between DNA methylation levels of each probe and host gene expression in colon adenocarcinoma tissues using MEXPRESS [13, 14], which is a web-based software that has DNA methylation data from The Cancer Genome Atlas (TCGA). Interestingly, DNA methylation levels of all four ZNF350 CpG sites were significantly and negatively correlated with *ZNF350* gene expression. In contrast, CpG sites of the other genes showed relatively weak or no significant correlation with their host gene expression (Table 2).

**Table 1:** List of the 35 hyper-methylated genes possibly associated with accelerated migration capacity in HCT116 cells

Gene symbol	Description	Methylation (%)			Expression (log fold)		Methy-lation sites	CpG context	Biofunction					Target ID
		Non-MG	MG	Delta methylation (MG - non-MG)	MG / non-MG	5-Azacytidine / vehicle			Cellular move-ment	Cellular develop-ment	Cellular growth and prolife-ration	Cell mor-phology	Cellular function and main-tenance	
*BEX5*	brain expressed X-linked 5	52.6	74.7	22.1	-1.90	1.13	TSS1500							cg05470074
*C2orf73*	chromosome 2 open reading frame 73	64.0	84.3	20.3	-0.72	1.37	Body	S_Shore						cg14794655
*CCDC149*	homo sapiens coiled-coil domain containing 149	53.5	75.8	22.3	-0.88	0.67	Body							cg24371114
*CCNG2*	cyclin G2	11.1	31.7	20.6	-1.05	0.94	5′UTR	S_Shore			**□**			cg19278744
*CLCN5*	chloride voltage-gated channel 5	30.4	57.7	27.3	-0.97	0.59	5′UTR	Island					**□**	cg02937293
		55.7	76.5	20.8			5′UTR	Island						cg07605754
		53.7	75.5	21.9			Body							cg07452499
*COL20A1*	collagen type XX alpha 1 chain	38.8	62.5	23.8	-0.81	0.88	Body	S_Shore						cg21391133
*CRABP1*	cellular retinoic acid-binding protein 1	50.4	73.3	22.9	-0.75	1.36	Body	S_Shelf						cg11592640
*CYYR1*	cysteine and tyrosine rich 1	57.8	79.2	21.4	-1.82	1.00	TSS200	S_Shore						cg21039874
*EFHD1*	EF-hand domain family member D1	11.7	37.2	25.5	-1.61	2.63	TSS1500	N_Shore						cg12118465
*ESPNL*	Espin-Like	41.8	66.4	24.6	-0.94	1.29	1stExon	N_Shore						cg01112020
*GPR37L1*	G protein-coupled receptor 37 like 1	54.3	76.9	22.6	-1.67	1.73	TSS200			**□**	**□**			cg15096829
		64.7	86.4	21.8			TSS200							cg17219660
*HLA-DPB2*	major histocompatibility complex, class II, DP beta 2 (pseudogene)	58.9	79	20	-0.66	0.62	Body							cg26650827
*IGFBP7*	insulin like growth factor binding protein 7	39.6	74.1	34.5	-2.39	2.15	Body		**□**	**□**	**□**			cg01684586
		33.7	64.7	31			Body							cg03984758
*KIAA1217*	KIAA1217	37.8	61	23.2	-2.12	1.68	5′UTR							cg05217312
		51.9	73.7	21.8			5′UTR							cg10116636
		54.5	77.4	23			5′UTR	S_Shelf						cg17646571
		55.9	77.2	21.3			5′UTR	S_Shore						cg26526047
*LAMA1*	laminin subunit alpha 1	37.7	62.3	24.6	-1.35	1.76	Body		**□**	**□**	**□**	**□**	**□**	cg18998321
*LRMP*	lymphoid restricted membrane protein	54.5	75.4	20.9	-1.40	2.51	TSS1500							cg03521113
*LTB*	lymphotoxin beta	50.4	73.1	22.7	-1.33	2.58	Body	Island	**□**		**□**	**□**	**□**	cg01106410
*LYPD1*	LY6/PLAUR domain containing 1	59	89.9	30.8	-1.48	0.89	1stExon	Island						cg25571189
*MAD2L2*	MAD2 mitotic arrest deficient-like 2 (Yeast)	11.6	32.6	21	-1.08	0.75	5′UTR	Island	**□**	**□**				cg25102735
*MMP25*	matrix metallopeptidase 25	61.3	87.4	26.1	-1.37	0.65	TSS200	Island						cg26927231
*NEO1*	neogenin 1	19.7	40.9	21.3	-1.30	0.69	Body	S_Shore	**□**	**□**	**□**		**□**	cg02356600
*OAS1*	2′-5′-oligoadenylate synthetase 1	16.8	54.5	37.7	-1.91	2.20	3′UTR							cg04708790
*OTP*	orthopedia homeobox	33.2	54.8	21.6	-0.77	2.57	5′UTR	N_Shore		**□**	**□**			cg24231716
*PAGE4*	PAGE family member 4	49.1	82.6	33.6	-0.88	0.63	TSS200							cg03675615
*PAX6*	paired box 6	57.4	78.2	20.8	-0.72	1.18	TSS200	Island	**□**	**□**	**□**	**□**	**□**	cg01867395
*PCSK6*	proprotein convertase subtilisin/kexin type 6	31.9	65.3	33.3	-1.14	0.62	Body		**□**					cg21122366
*PVRL4*	nectin cell adhesion molecule 4	52.7	74	21.3	-0.75	1.68	5′UTR							cg22585988
*RNASEH1*	ribonuclease H1	46.6	70.8	24.2	-0.81	1.52	Body	N_Shore						cg16363985
*SCN3B*	sodium voltage-gated channel beta subunit 3	55.6	75.8	20.2	-0.95	0.66	1stExon	Island			**□**			cg13765785
		7.3	30.2	22.9			Body							cg03112631
*SCNN1D*	sodium channel epithelial 1 delta subunit	56.1	81.8	25.7	-0.79	0.74	TSS1500							cg13587552
*SGPP2*	sphingosine-1-phosphate phosphatase 2	29.9	52.3	22.4	-1.70	0.93	TSS200	Island	**□**	**□**	**□**	**□**	**□**	cg23604012
*SLIT3*	slit guidance ligand 3	52.2	80.3	28	-1.45	1.48	Body	N_Shore	**□**			**□**		cg26119620
*SNORD24*	small nucleolar RNA, C/D box 24	31.1	58.2	27	-0.80	0.78	TSS200	S_Shore						cg20017995
*ZNF211*	zinc finger protein 211	49.3	71.8	22.4	-1.82	1.86	Body	Island						cg21262300
*ZNF350*	zinc finger protein 350	39.6	78.4	38.8	-3.78	2.57	5′UTR		**□**		**□**			cg02573825
		52.6	82.5	29.9			5′UTR							cg27565719
		30.2	50.9	20.7			TSS200							cg25782003
		27.5	76.9	49.3			TSS200							cg26498020

**Table 2:** Pearson’s correlation coefficient analysis between DNA methylation and host gene expression in colon adenocarcinoma tissues

Gene symbol	Target ID	Pearson’s r(DNA methylation vs gene expression)	*P* value
*BEX5*	cg05470074	-0.173	< 0.01
*C2orf73*	cg14794655	-0.00894	
*CCDC149*	cg24371114	-0.0623	
*CCNG2*	cg19278744	-0.113	
*CLCN5*	cg02937293	-0.131	
	cg07605754	-0.104	
	cg07452499	0.0375	
*COL20A1*	cg21391133	-0.0946	
*CRABP1*	cg11592640	0.328	< 0.001
*CYYR1*	cg21039874	-0.267	< 0.001
*EFHD1*	cg12118465	-0.284	< 0.001
*ESPNL*	cg01112020	-0.0784	
GPR37L1	cg15096829	-0.0399	
	cg17219660	-0.0498	
*HLA-DPB2*	cg26650827	N/A	
*IGFBP7*	cg01684586	-0.0479	
	cg03984758	0.169	< 0.05
*KIAA1217*	cg05217312	0.131	
	cg10116636	0.218	< 0.05
	cg17646571	0.0598	
	cg26526047	N/A	
*LAMA1*	cg18998321	0.257	< 0.001
*LRMP*	cg03521113	0.176	< 0.01
*LTB*	cg01106410	-0.115	
*LYPD1*	cg25571189	-0.203	< 0.05
*MAD2L2*	cg25102735	0.0905	
*MMP25*	cg26927231	-0.0899	
*NEO1*	cg02356600	-0.105	
*OAS1*	cg04708790	-0.0116	
*OTP*	cg24231716	0.0974	
*PAGE4*	cg03675615	0.178	< 0.05
*PAX6*	cg01867395	0.211	< 0.001
*PCSK6*	cg21122366	0.188	< 0.01
*PVRL4*	cg22585988	-0.0788	
*RNASEH1*	cg16363985	0.00651	
*SCN3B*	cg13765785	-0.154	< 0.05
	cg03112631	0.174	< 0.01
*SCNN1D*	cg13587552	0.0154	
*SGPP2*	cg23604012	-0.063	
*SLIT3*	cg26119620	-0.366	< 0.001
*SNORD24*	cg20017995	N/A	
*ZNF211*	cg21262300	-0.499	< 0.001
*ZNF350*	cg02573825	-0.736	< 0.001
	cg27565719	-0.76	< 0.001
	cg25782003	-0.693	< 0.001
	cg26498020	-0.676	< 0.001

**Figure 6:**
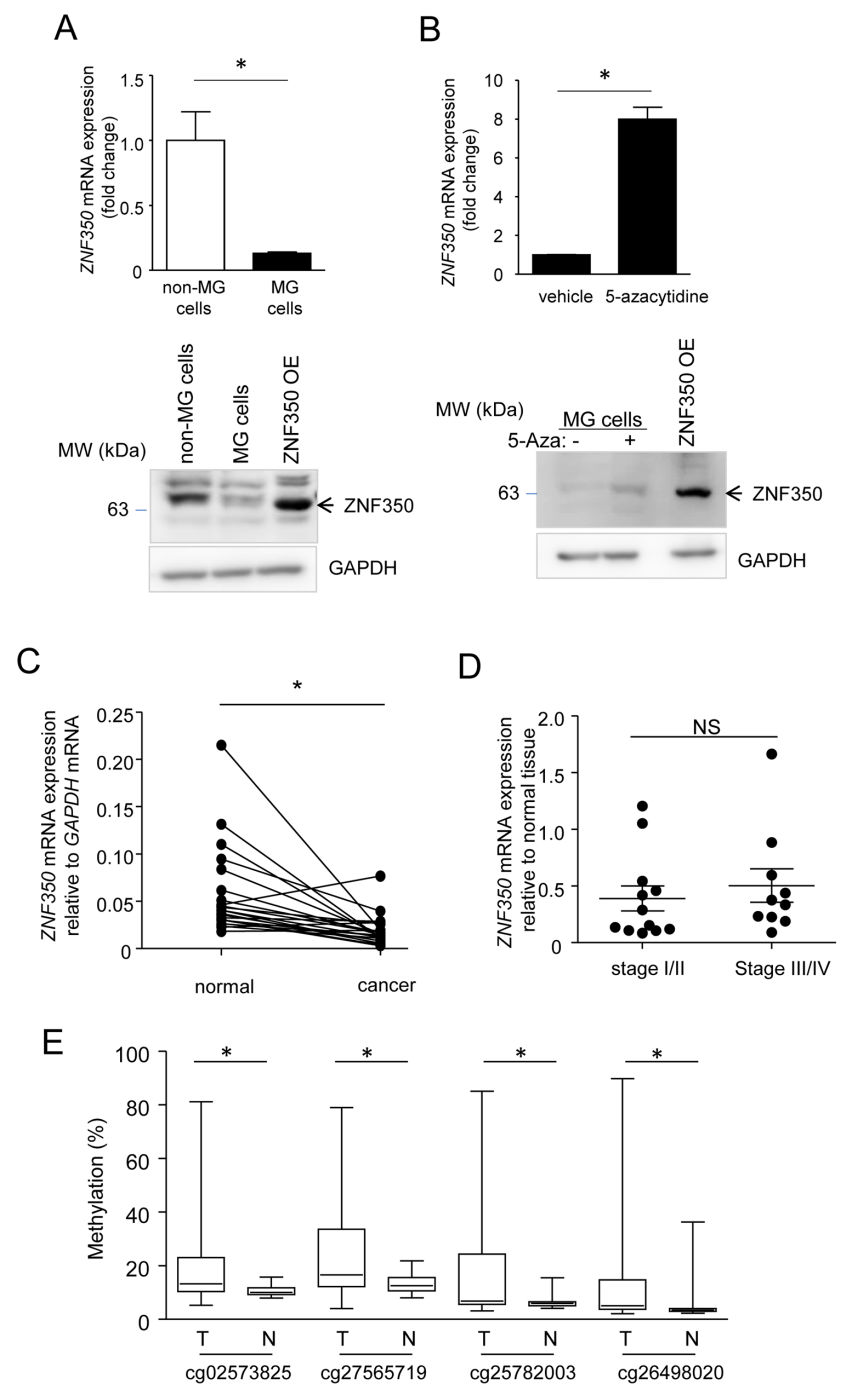
Down-regulated expression of *ZNF350* mRNA in MG cells and colorectal cancer **(A)** qPCR analysis of *ZNF350* mRNA expression. The data were normalized to *GAPDH* mRNA levels and are expressed as the mean fold change ± SD (n = 4) compared to that in non-MG cells. ^*^P < 0.01, unpaired Student’s *t*-test. **(B)** *ZNF350* mRNA levels in the MG cells after treatment with 5 μM 5-azacytidine or vehicle. Data are expressed as the mean fold change ± SD (n = 4) compared with the vehicle-treated MG cells. ^*^P < 0.01, unpaired Student’s *t*-test. **(C)** Using qPCR, we measured the expression of *ZNF350* mRNA in cDNA libraries prepared from colon cancers and surrounding normal colonic mucosa from 22 patients. ^*^P < 0.01, Wilcoxon signed rank test. **(D)** We compared the expression of *ZNF350* mRNA relative to the surrounding normal tissues in Stage I/II of colon cancers with those in stage III/IV. NS; no significant difference by the unpaired Student’s *t*-test. **(E)** DNA methylation of CpG sites in *ZNF350* promoter in cancer tissues (T) was compared with that in normal tissues (N), using a web-based software, MethHC. CpG sites were indicated as ‘cgxxxxxxxx’, which are target IDs of the Illumina HumanMethylation 450 BeadChip array. ^*^P < 0.001 by the unpaired Student’s *t*-test.

Based on these data, we were particularly interested in *ZNF350* encoding zinc finger protein 350 (ZNF350/ZBRK1) that has been suggested to function as a tumor suppressor, namely repression of metastasis/invasion, via interaction with breast cancer 1 (BRCA1) and KRAB-ZFP-associated protein 1 (KAP1) [15]. Notably, *ZNF350* mRNA expression in the MG cells was reduced to 13% compared to that in non-MG cells (Figure 6A), and treatment with 5-azacytidine increased its expression by 8-fold (Figure 6B). Furthermore, we examined *ZNF350* expression in cDNAs prepared from paired normal and tumor tissues of 22 patients with colon adenocarcinoma (HCRT103 TissueScan qPCR Arrays; OriGene Technologies, Rockville, MD, USA), and found that colon cancer tissues have significantly reduced *ZNF350* mRNA expression when compared with the surrounding normal colon tissues (Figure 6C). However, there was no significant difference in relative changes in *ZNF350* mRNA levels (cancer/normal) between stages I/II and stages III/IV colon cancers (Figure 6D). In addition, a web-based software, MethHC, showed that all four CpG sites of ZNF350 promoter were significantly hyper-methylated in colon cancer tissues compared with normal tissues (Figure 6E).

### Regulation of cell migration by ZNF350

**Figure 7:**
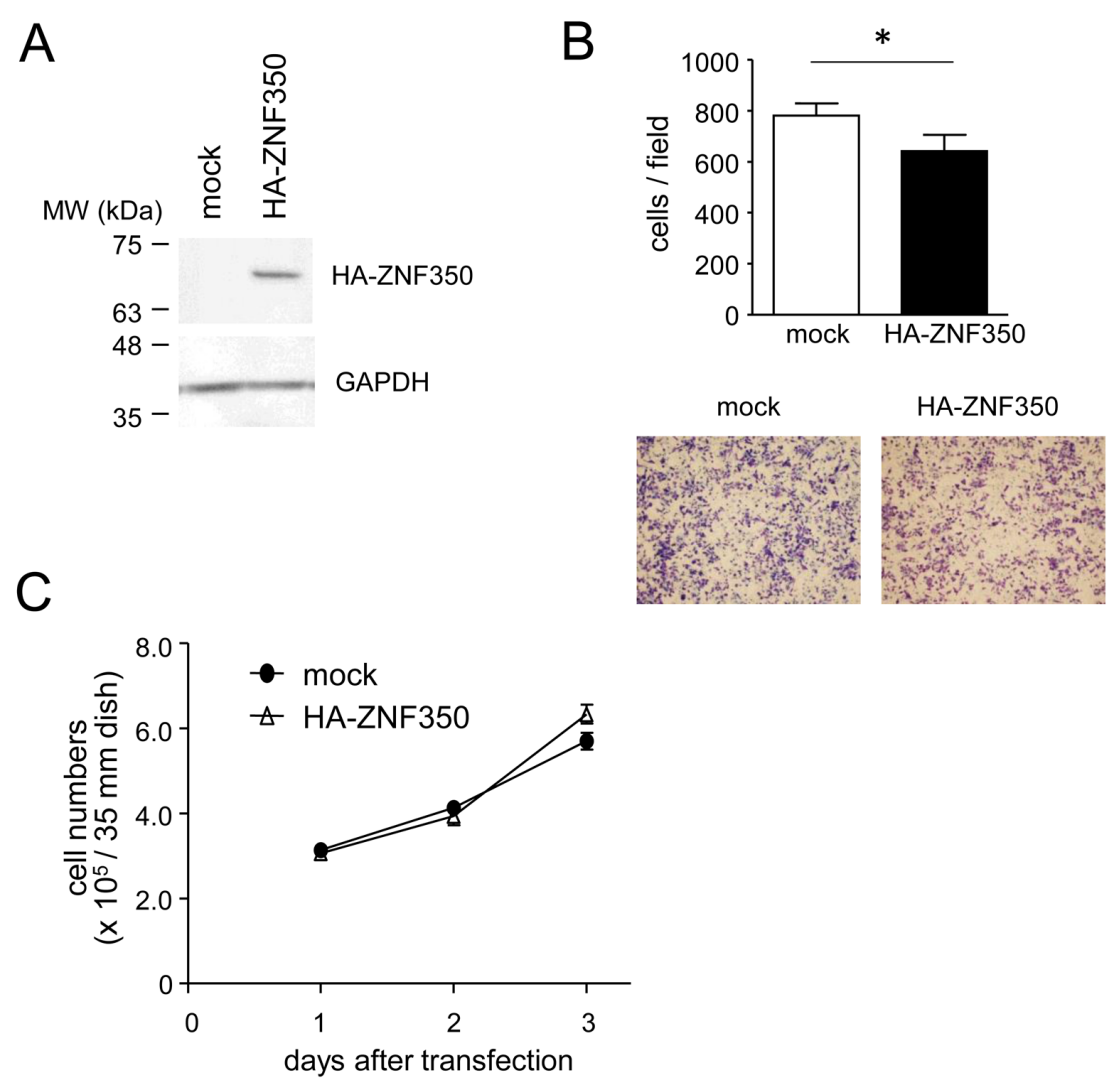
Loss of migratory capacity in ZNF350-overexpressing MG cells **(A)** Western blot analysis of ZNF350 overexpression in MG cells. MG cells were transfected with either an empty (mock) or a HA-ZNF350-expression vector. GAPDH levels were used as an endogenous quantitative control. **(B)** Effects of ZNF350 overexpression on cell mobility. After the MG cells were transfected with either an empty (mock) or a HA-ZNF350-expression vector, they were seeded in serum-free medium on the upper side of a transwell chamber and allowed to migrate towards 10% FBS-containing medium in the lower chamber. After incubation for 24 h, migrating cells were subjected to Diff-Quick staining (lower panels) and counted (upper panel). Data are presented as the means ± SD (n = 4). ^*^P < 0.05, unpaired Student’s *t*-test. **(C)** Analysis of growth rate in MG cells overexpressing ZNF350. Data are presented as the means ± SD (n = 4). MW, molecular weight.

**Figure 8:**
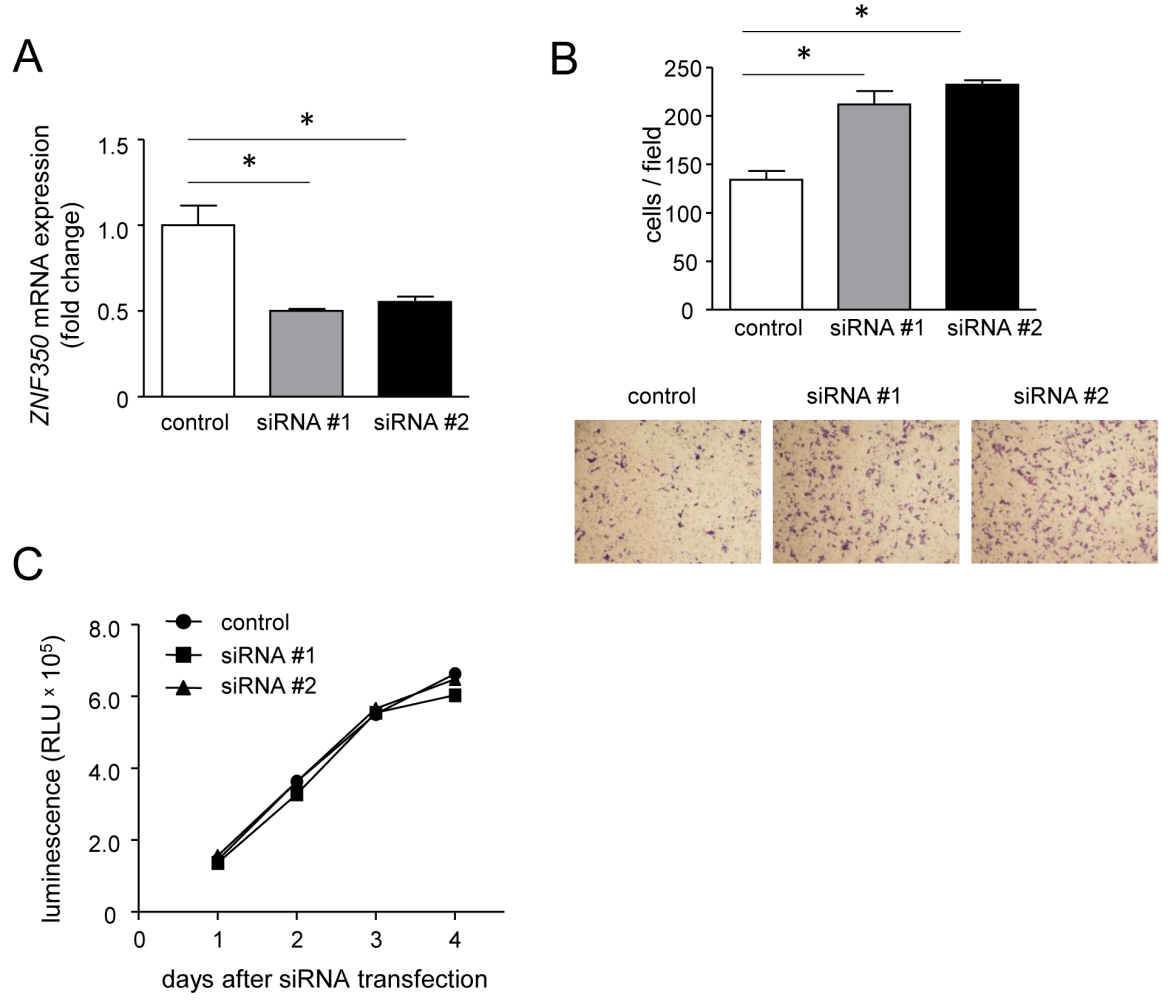
Increase of migratory capacity in non-MG cells after ZNF350 knockdown **(A)** *ZNF350* gene silencing using two siRNAs (siRNA#1 and #2) or RNAi-negative control was confirmed by qPCR. Data are expressed as the mean fold changes ± SD (n = 4) compared with those in control siRNA-treated non-MG cells. ^*^P < 0.01, unpaired Student’s *t*-test. **(B)** Migration assay after the treatment of non-MG cells with one of the two different ZNF350 siRNAs (siRNA#1 and #2) or RNAi-negative control. The migrating cells were subjected to Diff-Quick staining (lower panels) and counted (upper panel). Data are presented as the means ± SD (n = 4). ^*^P < 0.01, unpaired Student’s *t*-test. **(C)** Analysis of growth rate in the non-MG cells treated with siRNA at the indicated times. Data are presented as the means ± SD (n = 4).

To confirm that ZNF350 plays a significant role in the altered migratory capacity of HCT116 cells, we prepared MG cells that transiently overexpress ZNF350 as well as non-MG cells with silenced ZNF350. ZNF350 overexpression in MG cells (Figure 7A) resulted in a small, but significant, reduction in migration (Figure 7B) without affecting cell growth (Figure 7C). In contrast, ZNF350 silencing in non-MG cells (Figure 8A) significantly accelerated their migration (Figure 8B) without affecting their growth (Figure 8C).

### Identification of hyper-methylated CpG sites within the *ZNF350* promoter

**Figure 9:**
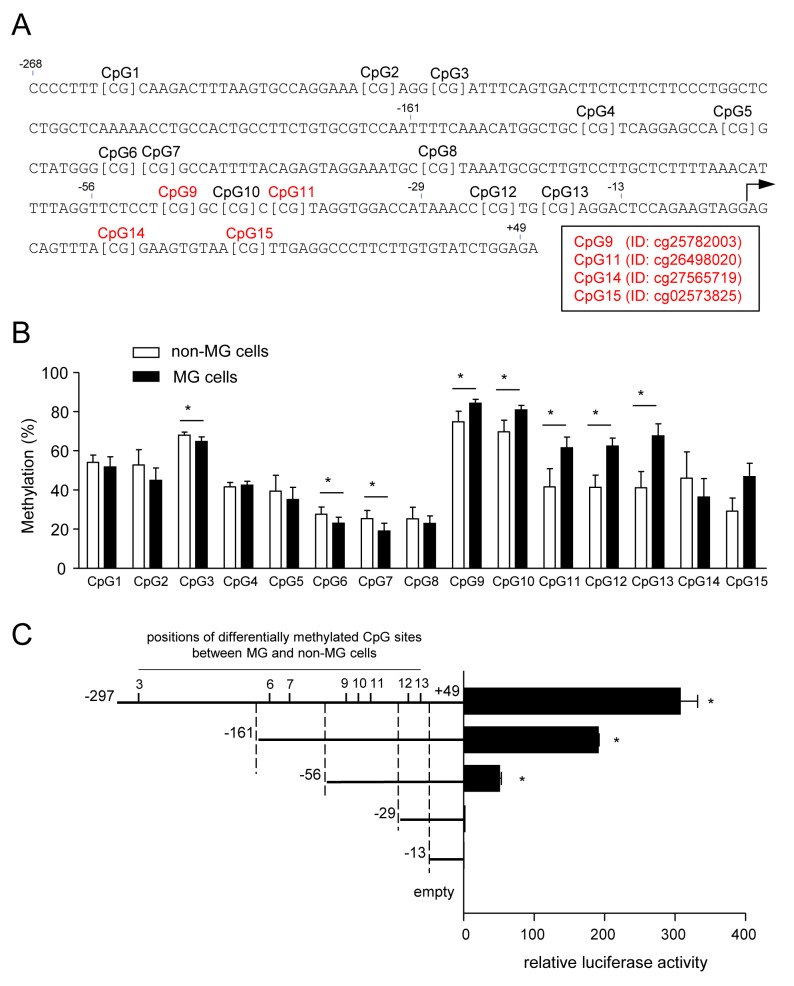
Methylation of CpG sites in the *ZNF350* promoter **(A)** Pyrosequencing analysis predicted 15 possible methylation sites in the proximal promoter region of *ZNF350*. Probes (target IDs) carried on the Infinium HumanMethylation450 BeadChip are indicated in red. **(B)** The percentage of DNA methylation in MG and non-MG cells was analyzed via highly quantitative bisulfite pyrosequencing. Data are presented as the means ± SD (n = 4). ^*^P < 0.05, unpaired Student’s *t*-test. **(C)** Dual-luciferase reporter assay showing the changes in *ZNF350* promoter activity after the specified truncation in the non-MG cells. Data are presented as the means ± SD (n = 4). ^*^P < 0.01, unpaired Student’s *t*-test compared to control (empty).

Finally, we sought to identify the hyper-methylated CpG sites within the *ZNF350* promoter responsible for its transcriptional down-regulation in the MG cells. The Infinium HumanMethylation450 BeadChip carries probes for 16 CpG sites in *ZNF350* (-1,036 bp from the transcription starting point to intron 4). The pyrosequence analysis software predicted 15 possible methylation sites in the proximal promoter region (Figure 9A). The Infinium HumanMethylation450 BeadChip revealed four hyper-methylated CpG sites in the *ZNF350* promoters of the MG cells: CpG 9 (Target ID, cg25782003), CpG11 (cg26498020), CpG14 (cg27565719), and CpG 15 (cg02573825), when a difference greater than 20% was established as significant. There were no significant differences in the methylation status at any of the other CpG sites between the MG cells and non-MG cells. Pyrosequencing confirmed that CpG9, 10, 11, 12 and 13 were hyper-methylated, while CpG3, 6, and 7 were hypo-methylated to a lesser extent in the MG cells, compared to the methylation status of these sites in non-MG cells (Figure 9B).

We next examined the importance of the promoter regions containing the hyper-methylated sites in *ZNF350* promoter activity. The relative luciferase activities of serially truncated fragments showed that the -297 to -161 bp, -161 to -56 bp, and -56 to -29 bp fragments had an effect on *ZNF350* promoter activity (Figure 9C). These data suggest that hyper-methylation of the *ZNF350* promoter at CpG9, 10, and 11 may play a significant role in the down-regulation of *ZNF350* transcription in the MG cells, leading to increased migration.

## DISCUSSION

Intra-tumor heterogeneity is a fundamental mechanism used by cancers to cope with diverse surrounding microenvironments. This heterogeneity is a result of transcriptomic, epigenomic, proteomic, metabolic, and functional diversification, all of which may occur during expansion of the neoplastic cell population [16–18]. Even cultured tumor cell lines are known to be highly heterogeneous [19]. However, the role of DNA methylation and the specific genetic differences between these cell populations are largely unknown.

In this study, we successfully isolated highly motile cells and control immotile cells from the colon cancer cell line HCT116 using a transwell-based method. Compared with the immotile subpopulation, the highly motile cells possessed some signature mesenchymal gene markers, including increased *ZEB1* and *VIM* expression along with their positive regulators. In contrast, EMT-associated down-regulation of *CDH1* and up-regulation of *SNAIL1* was not observed. These data suggest that the MG cells may be in a hybrid EMT state, also known as a metastable state or partial EMT, in which individual cells express both epithelial and mesenchymal markers. Notably, this is commonly observed in carcinomas [20]. Although the process of EMT, either as a gradual, continuous change or by discrete stages, is highly debated in the literature, the presence of distinct, stable, long-lasting cell populations suggests that EMT progresses via discrete intermediate stages [20, 21]. Recently, Pastushenko *et al.* investigated intra-tumor diversification in organoids derived from multiple single cells isolated from colorectal cancers and characterized six distinctive cell subpopulations [22]. They also identified two subpopulations, both of which were in an intermediate EMT state, that represent most metastatic cells. These EMT hybrid cells are likely present *in vivo* as stable cell populations [22]. Since cell migration plays a pivotal role during invasion and metastasis in various types of tumors [1, 2], we sought to characterize our MG cells. Notably, cell growth was similar for the MG and non-MG cells, and their cell migration phenotypes and gene expression patterns remained constant even after five passages under our standard culture conditions. These data suggest that the isolated motile phenotype may already exist in the original parent HCT116 cells as one of its heterogeneous subpopulations.

Colorectal cancer cells experience a substantially higher rate of somatic mutations than normal colorectal cells, particularly during the final dominant clonal expansion of the cancer [22]. Mutation-related genetic diversification within cancer cell populations is also observed as a common feature of colorectal cancers [22]. Additionally, epigenomic diversification may also occur during expansion of the neoplastic cell population [16–18]. In fact, aberrant methylation is known to be involved in the abnormal expression of genes that regulate critical cancer cell processes, such as proliferation, apoptosis, migration, and/or invasion. This mechanism is well-illustrated by the hyper-methylation of the *RB1* promoters in retinoblastoma [7]. Silencing of the *BRCA1* gene by promoter hyper-methylation has also been detected in primary breast and ovarian carcinomas, especially during loss of heterozygosity and in specific histopathologic subgroups [10]. Furthermore, hyper-methylation of the *hMLH1* promoter has been shown to cause gene inactivation in colorectal cancers as well as widespread microsatellite instability [8, 9]. Indeed, the presence or absence of *MLH1* methylation has been suggested to critically affect the heterogeneity of colorectal carcinoma [23].

In this study, the motile MG cells completely lost their motility following de-methylation with 5-azacytidine, which resulted in the up-regulation of 1,552 related genes. Combining the differentially expressed genes and DNA methylation profile of the MG cells with their gene expression signatures after 5-azacytidine treatment identified 35 hyper-methylated putative target genes responsible for the observed acquisition of motility in the HCT116 cells. We found the hyper-methylation of the *ZNF350* promoter and further showed that it significantly affects the characteristics of the colon cancer cells, especially migration. Moreover, *ZNF350* mRNA levels in the MG cells were reduced to 13% of those observed in non-MG cells, and 5-azacytidine treatment completely recovered *ZNF350* mRNA expression and ZNF350 protein levels. Interestingly, ZNF350 tends to be underexpressed in colon cancers [24]. Consistent with this previous work, we found that colon cancer tissues had significantly reduced *ZNF350* mRNA levels compared to the surrounding normal colon tissues. Considering the possible role of ZNF350, one might speculate that the larger cancer and more advanced cancer should show lower levels of ZNF350. However, no significant stage-dependent change in *ZNF350* mRNA expression was observed. In addition, the analysis of TCGA data using MEXPRESS showed that there was no significant correlation between *ZNF350* mRNA expression and pathologic stage of colon adenocarcinoma (Pearson’s r = + 0.0549). ZNF350-overexpressing MG cells had a small, but significant inhibitory effect on migratory activity, while *ZNF350*-silenced non-MG cells had accelerated migration. Thus, methylation of the *ZNF350* promoter may be one of the crucial determinants for the acquisition of motility during colon cancer pathogenesis.

As shown in Figure 7, overexpression ZNF350 protein had a limited effect on migration, suggesting that other regulators should be involved in the acquisition of the migratory phenotype. On the other hand, in siRNA experiments, both ZNF350 siRNA #1 and #2 reduced its levels only by 50%. Other commercially available siRNAs showed similar knockdown efficacy (data not shown). However, even 50% reduction facilitated migration of immotile cells. Although our results suggest that ZNF350 may be one of the crucial factors for the acquisition of motility in HCT116 cells, multiple key regulators are likely involved in the alteration of phenotype.

ZNF350, also known as zinc-finger and BRCA1-interacting protein with a Kruppel-associated box (KRAB) domain (ZBRK1), is involved in the development of several human tumor types, including breast, colon, and cervical carcinomas [24–26]. ZNF350 contains an N-terminal A+B box domain, eight C_2_H_2_ zinc fingers, and a C-terminal repression domain (CTRD) [27, 28]. The CTRD forms tetrameric oligomers that allow ZNF350 to selectively interact with BRCA1, some histone deacetylases, and certain gene promoters [29, 30], including those of *GADD45A* [27], *ANG1* [31], *HMGA1* [32], *p21* [33], *MMP9* [26], *FGF2* [34], *SNAI2* [35] and *KAP1* [29, 36], leading to transcriptional repression. In cervical cancer, increased *ZNF350* gene expression is correlated with inhibition of growth and metastasis of tumor cells. ZNF350 is also known to inhibit cervical carcinoma metastasis, perhaps via modulation of MMP9 and KAP1 expression [29, 36]. In addition, ZNF350 acts as a transcriptional corepressor with nuclear villin and represses Slug (*SNAI2*) expression, resulting in inhibition of EMT [35]. Among the ZNF350-regulated, EMT-related genes (*MMP9*, *KAP1*, and *SNAI2*), *SNAI2* expression was upregulated in the MG cells, and ZNF350 overexpression repressed *SNAI2* expression, suggesting that *SNAI2* may be one of the potential targets (Supplementary Figure 2). A possible mechanism is that the ZNF350/BRCA1/CtTB-interacting protein complex represses the expression of angiopoietin-1 (ANG1) and high-mobility group AT-hook 2 (HMGA2), which are commonly involved in the proliferation and vascularization during breast tumor formation [31, 32]. At present, however, there is no direct evidence that ZNF350/BRCA1 complex represses transcriptional activity of migratory phenotype-related genes in the MG cells. The IPA analysis showed that several BRCA1-regulated genes, such as *BCL2*, *VIM*, *NFE2L2 and IFITM1*, were differently expressed in the MG cells (Supplementary Figure 3). These genes might be involved in acquisition of migratory capability in the MG cells through ZNF350/BRCA1 complex. Although further studies are needed to figure out how ZNF350 works with BRAC1 complex to repress migration, our results suggest that ZNF350 could also be a potential tumor suppressor in different types of cancers, including colorectal cancer.

To better understand the regulation of *ZNF350* in the highly mobile HCT116 cells, we performed a pyrosequencing analysis of 15 CpG sites in the promoter region of this gene (from -297 to +14 bp), and succeeded in detecting several significant difference in endogenous methylation state; three hypo-methylated and five hyper-methylated CpG sites in the MG cells. We prepared serially truncated fragments of the proximal promoter of *ZNF350* and measured their reporter activities. When we considered the distribution of differentially methylated CpG sites between the MG and non-MG cells on the promoter fragments, the fragment containing hypo-methylated CpG sites (CpG 3, 6 and 7) or hyper-methylated CpG sites (CpG 9, 10 and 11) possessed the promoter activity. We focused on the DNA methylation-dependent mechanism for the down-regulated expression of ZNF350, since treatment with 5-azacytidine decreased migration capacity in association with an increase in *ZNF350* mRNA levels. Moreover, MEXPRESS indicated that DNA methylation levels of ZNF350 were significantly and negatively correlated with *ZNF350* gene expression. In general, hypo-methylated CpG sites are associated with transcriptional activation. Therefore, we did not investigate a role of hypo-methylated CpG 3, 6, and 7 in the MG cells, although these hypo-methylated sites might also play a role in the transcriptional activity of *ZNF350* mRNA. CpG 12 and 13 were hyper-methylated in the MG cells. However, the fragment containing these sites did not show significant promoter activity. A web-based software, TFBIND [37], predicts that the sequences (-18 to -32) including CpG 12 and 13 may contain a TATA-box sequence, which determines a transcription initiation sites. Therefore, we have to consider the possible role of CpG 12 and 13 in the transcription initiation of *ZNF 350*. At present, however, we could not directly assess the role of specific methylation sites in the promoter activity by the transient transfection method used in our experiments. Further studies are needed to clarify the mechanism. Although there are still several concerns and limitations, our results suggest that the hyper-methylation of three CpG sites (CpG 9, 10, and 11) in the *ZNF350* promoter may function as putative methylation sites involved in the down-regulation of gene expression and accelerated cellular migration. These data are supported by a previous study showing that *ZNF350* and *MAGED1* were hyper-methylated in tamoxifen-resistant breast cancer cell lines, and treatment with 5-aza-2′deoxycytidine caused a significant reduction in promoter methylation of both genes [38].

In this study, we focused on a particular protein, ZNF350, because ZNF350 showed the highest correlation between DNA methylation and altered gene expression in clinical samples (TCGA data set) (Table 2). We could identify ZNF350 as one of the crucial molecules involved in the acquisition of migratory capability through DNA methylation. However, distinct genes included in Table 1 and others should be also involved in the process. Indeed, among the listed genes in Table 1, several gene products, such as IGFBP7, MAD2L2, and SLIT3 are known to have inhibitory functions on migration. A DNA methylation inhibitor attenuated migration ability through induction of IGFBP7 in colon cancer cell lines [39], and IGFBP7 could inhibit cell growth of breast tumor cells [40]. MAD2L2 interacted with NCOA3 and suppressed proliferation and migration of colon cancer cells [41]. SLIT3 was also induced by a DNA methylation inhibitor [42], and silencing of SLIT3 promoted proliferation, migration, and invasion with enhanced expression of MMP2 and MMP9 in lung cancer cells [43]. Our genome-wide analyses of DNA methylation and gene expression profiles identified ZNF350 as a DNA methylation-dependent regulator, which may determine a cancer phenotype among heterogeneous subpopulation. At present, however, we can not conclude that ZNF350 is a key regulator for motility of colon cancer cells. Moreover, we particularly focused on the motility in this study. Different states of EMT require the concerted efforts of many factors.

In conclusion, we analyzed the role of DNA methylation in the regulation of genes functioning in a highly mobile subpopulation of HCT116 cells. Our results indicate that these hybrid cells possessed hyper-methylated CpG sites in the proximal promoter of *ZNF350*. Furthermore, we provide evidence that hyper-methylation of the *ZNF350* promoter may be one of the crucial determinants for the acquisition of increased migratory capabilities in colon cancer cells. To our knowledge, this is the first study to investigate the relationship between CpG methylation and a migratory phenotype in a heterogeneous colon cancer cell population. While further studies are needed to fully understand the potential role of *ZNF350* promoter methylation in the process of colon cancer metastasis, the present study enhances our understanding of heterogeneous cell populations during carcinogenesis and highlights an essential role for DNA methylation.

## MATERIALS AND METHODS

### Isolation of a subpopulation with high cell migration capacity

The colon cancer cell line HCT116 was cultured in Dulbecco’s modified Eagle’s medium (DMEM; Nacalai Tesque, Kyoto, Japan), supplemented with 10% (vol/vol) heat-inactivated fetal bovine serum (FBS) at 37°C in 5% CO_2_. For isolation of the highly migratory subpopulation, we established the following selection procedures using a transwell migration assay modified from the Boyden chamber assay [12]. After serum starvation for 48 h, the HCT116 cells were seeded in serum-free medium on the upper side of a transwell chamber (Becton Dickinson, Franklin Lakes, NJ, USA) and allowed to migrate towards the chamber with DMEM containing 10% FBS. After 48 h, the non-MG cells remaining on the upper membrane and MG cells migrating onto the lower side of the membrane were collected separately using a cell detachment solution (Accutase™, Nacalai Tesque). Both non-MG and MG cells collected from the upper and lower chamber, respectively, were then separately seeded and cultured with 10% FBS. The resulting MG and non-MG cell types were further concentrated using another transwell migration assay. This selection was repeated 5 times to purify the MG and non-MG cell populations.

### Cell growth and migration assays

Cell growth was assessed by counting the number of cells with a hematocytometer or by the Cell Titer-Glo assay (Promega, Madison, WI, USA). Cell migration was examined using 8 μm pore size polycarbonate transwell filters (Becton Dickinson). After serum starvation for 48 h, the cells were seeded in serum-free media on the upper side of a transwell chamber and allowed to migrate towards media containing 10% FBS in the lower chamber for 24 h. After migration, the cells on the lower side of the membrane were fixed, stained with Diff-Quick stain (Sysmex, Kobe, Japan), and counted. The migration indices were calculated as the mean number of cells in 5 random fields at 20× magnification.

### Small interference RNAs (siRNAs)

To silence *ZNF350* expression, two siRNAs were designed (Supplementary Table 1) (Nippon Gene, Tokyo, Japan). RNAi-negative control (Nippon Gene) was used as a control siRNA. Cells were treated with the indicated siRNAs at a final concentration of 20 nM using Lipofectamine RNAiMAX (Invitrogen, Carlsbad, CA, USA) by the reverse transfection method according to the manufacturer’s protocol.

### Plasmid construction and cell transfection

A cDNA library was prepared from HCT116 cells, and the ZNF350 open reading frame (Ensembl Transcript ID: ENST00000243644) was amplified by PCR using the primer set listed in Supplementary Table 1. The amplified products were separated with a gel extraction kit (Qiagen) and cloned into the mammalian expression vector pEBMulti-puro (Wako, Osaka, Japan). An HA tag was appended to the C-terminus of the insert sequence. The construct sequence was confirmed by DNA sequencing. The MG cells were then transfected with either empty or HA-ZNF350-expression vector using Xtreme HP reagent (Roche, Basel, Switzerland) according to the manufacturer’s protocol.

### Global DNA methylation analysis

Genomic DNA was extracted from the MG and non-MG cells, and bisulfate-converted DNA was prepared using an EZ DNA methylation-gold kit (Zymo Research, Irvine, CA, USA). The bisulfate-converted DNA was then subjected to global DNA methylation analysis using an Infinium HumanMethylation450 BeadChip array (Illumina) as highlighted in Figure 4. The resulting methylation data were processed using GenomeStudio Methylation Module v1.9.0 software (Illumina). For quality control, only methylation measures with a detection *P* value less than 0.05 were used. The methylation levels of CpG sites were then calculated as β-values using the GenomeStudio methylation software after color balance adjustment and background corrections in the same chip. All of the Illumina methylation 450 data have been deposited in NCBI Gene Expression Omnibus (GEO; accession number GSE114683).

### Gene expression profiling

Total RNA was extracted from cells using an RNeasy kit (Qiagen) according to the manufacturer’s protocol. Concentration and purity of the extracted RNA were determined with a NanoDrop ND-1000 spectrophotometer (NanoDrop Technologies, Wilmington, DE, USA). Quality of the purified RNA was also assessed with an Agilent 2100 Bioanalyzer using an RNA 6000 Nano Labchip kit (Agilent). RNA samples with an RNA integrity number greater than 9.0 were used for further analysis. The mRNA expression profiles were then evaluated using a human mRNA microarray (SurePrint G3 Human; Agilent). The data were analyzed using GeneSpring 14.9 (Agilent). Notably, we eliminated mRNA signals within the lowest 20th percentile of all the intensity values in at least half of the samples and filtered the data set on existing flag values. Consequently, 24,189 probes (21,344 genes) in total were detected in our samples. The microarray and sample annotation data have been deposited in GEO (accession number GSE114681).

### Measurement of DNA methylation by pyrosequencing

We also analyzed genomic DNA methylation in the MG and non-MG cells by pyrosequencing (Qiagen). PCR and sequencing primers were designed using PyroMark Assay Design 2.0 software (Qiagen). Pyrosequencing procedures were performed according to the manufacturer’s protocol. Regions of the *ZNF350* promoter (region 1, -324 to -200 nt; region 2, -223 to -56 nt; region 3, -205 to +72 nt; and region 4, -17 to +72 nt from the transcription start site) were separately amplified by PCR using the primer sets shown in Supplementary Table 2. Bisulfite-converted genomic DNA (500 ng) was prepared using an EpiTect kit (Qiagen). The converted DNA was then amplified by PCR using a PyroMark PCR Master Mix kit (Qiagen). Then, the biotinylated PCR products were immobilized onto streptavidin-coated beads (GE Healthcare, Piscataway, NJ, USA), and the DNA strands were separated using the PyroMark denaturation solution (Qiagen). After washing and neutralization using a PyroMark Q24 Vacuum Workstation, the sequencing primer was annealed to the immobilized strand. DNA methylation was analyzed via highly quantitative bisulfite pyrosequencing with a PyroMark Q24 system (Qiagen). Data were analyzed using PyroMark Q24 software (Qiagen) to determine the methylation level (calculated as a percentage of methylation at each CpG).

### Quantitative real-time reverse transcription-PCR (qPCR)

Total RNA was extracted from cells using RNA iso plus reagent (Takara, Otsu, Japan). The isolated RNA (1 μg) was then reverse-transcribed using ReveTra Ace qPCR RT Master Mix (Toyobo, Osaka, Japan). *CDH1, ZEB1, SNAIL1, VIM,* and *ZNF350* mRNA levels were measured using SYBR Green Master Mix with an Applied Biosystems 7500 Real-time System (Applied Biosystems, Foster City, CA, USA). The sequences of the primer sets are listed in Supplementary Table 1. The target mRNA levels were calculated uding the comparative ΔΔCt method with glyceraldehyde 3-phosphate dehydrogenase (*GAPDH*) mRNA as an endogenous quantitative control. The expression of each gene is presented as the relative change compared to that in the indicated control sample.

Further, a TissueScan qPCR Array (HCRT103), which includes cDNAs from the paired normal and tumor tissues of 22 patients with colon adenocarcinoma (5 cases in stage I, 7 cases in stage II, 8 cases in stage III, and 2 cases in stage IV), was obtained from OriGene Technologies. Using this array, ZNF350 mRNA levels in human colon adenocarcinoma were measured by qPCR and normalized to those of *GAPDH* mRNA.

### Methylation modifications analysis of clinical samples

To investigate the correlation between DNA methylation and host gene expression, colon adenocarcinoma (COAD) samples in MEXPRESS datasets were analyzed [13]. We then used MethHC to compare DNA methylation of the *ZNF350* promoter in colon adenocarcinoma tissues with that in normal tissues [44].

### Western blotting

Whole-cell lysates were prepared using RIPA buffer (10 mM Tris-HCl, pH 7.4; 1% Nonidet P-40; 1 mM EDTA; 0.1% SDS; 150 mM NaCl) containing a protease inhibitor cocktail (Nacalai Tesque) and a phosphatase inhibitor cocktail (Sigma). The extracted proteins were separated by sodium dodecyl sulfate (SDS)-polyacrylamide gel electrophoresis and then transferred onto a polyvinylidene difluoride (PVDF) membrane (Bio-Rad, Hercules, CA). After blocking for 1 h at room temperature with 5% nonfat milk (Cell Signaling Technology, Danvers, MA), the membrane was incubated overnight at 4°C with a rabbit polyclonal anti-HA antibody (1:2000; MBL, Nagoya, Japan) or anti-ZNF350 antibody (1:1000; Abcam, Cambridge, CA, USA). Following incubation with an appropriate secondary antibody for 1 h at room temperature, bound antibodies were detected with an ECL Prime Western Blotting Detection System (GE Healthcare). The PVDF membrane was then re-blotted using a mouse monoclonal anti-GAPDH antibody (1:5000; Santa Cruz Biotechnology, Santa Cruz, CA, USA). Intensities of the bound antibodies were quantified using Image J software.

### Promoter activity assay

Serially truncated segments (-297 to +49, -161 to +49, -56 to +49, -29 to +49, and -13 to +49 bp) of the *ZNF350* proximal promoter region were amplified using the primer sets listed in Supplementary Table 1. The first PCR was performed using human genomic DNA as a template. The amplified products were separated with a gel extraction kit (Qiagen) and cloned into the pGL4.21-basic vector (Promega) using XhoI and HindIII restriction sites. HCT116 cells (1.0 × 10^5^) were cultured on 24-well plates, and pGL-4.21 luciferase constructs with the various segments (100 ng) were co-transfected with the pGL4.74 vector (50 ng) using X-tremeGENE HP DNA transfection reagent (Roche). Then, 24 h after the transfection, the cells were harvested, and the firefly and Renilla luciferase activities were measured using a Dual-Luciferase Reporter Assay System (Promega).

### Statistical analysis

All statistical analyses were performed with GraphPad Prism 5 software and Microsoft Excel. Results are expressed as the means ± standard deviation (SD). Significant differences between two groups were estimated by two-tailed Student’s *t-*test. Non-parametric data were analyzed using the Wilcoxon signed rank test. *P* values less than 0.05 were considered statistically significant.

## SUPPLEMENTARY MATERIALS FIGURES AND TABLES



## Abbreviations

EMT: epithelial-mesenchymal transition
CG: cytosine-guanine
UV: ultraviolet
MG cells: migrated cells
IPA: ingenuity pathway analysis
CTRD: C-terminal repression domain
DMEM: Dulbecco’s modified Eagle’s medium
FBS: fetal bovine serum
HA: hemagglutinin
PCR: polymerase chain reaction
QPCR: quantitative real-time reverse transcription-PCR
PVDF: polyvinylidene difluoride
