# Supplementary material for: ZNF350 promoter methylation accelerates colon cancer cell migration
Source: Oncotarget. 2018 Dec 4;9(95):36750–69. doi: 10.18632/oncotarget.26353 (PMC6298409; doi:10.18632/oncotarget.26353)
Supplement: Supplementary file 1 [file oncotarget-09-36750-s001.pdf]

ZNF350 promoter methylation accelerates colon cancer cell migration

SUPPLEMENTARY MATERIALS

Supplementary Table 1: Primer sets used for qPCR and cloning ZNF350 sequence, and oliginucleotide sequences for siRNA

**Primers for qPCR**		
**Targets**		**primer sequences (5′-3′)**
CDH1	forward	TGGAGGAATTCTTGCTTTGC
	reverse	CGCTCTCCTCCGAAGAAAC
SNAIL1	forward	GCTGCAGGACTCTAATCCAGA
	reverse	ATCTCCGGAGGTGGGATG
ZEB1	forward	GGAGGATGACACAGGAAAGG
	reverse	TCTGCATCTGACTCGCATTC
VIM	forward	TGTCCAAATCGATGTGGATGTTTC
	reverse	TTGTACCATTCTTCTGCCTCCTG
ZNF350	forward	TCTTGTGTATCTGGAGAAAATAGAGGT
	reverse	AAGAAATGGTGAACCCCAAA
GAPDH	forward	AGCCACATCGCTCAGACAC
	reverse	GCCCAATACGACCAAATCC
Primers for *ZNF350* promoter cloning		
ZNF350 (-297)	forward	AAAAACTCGAGTGATAAAGCCTGAGTCTCTGAAAATCTGC
ZNF350 (-161)	forward	AAAAACTCGAGTTTCAAACATGGCTGCCGTCAGGAGC
ZNF350 (-56)	forward	AAAAACTCGAGTTCTCCTCGGCCGCCGTAGGTGGACCATAAAC
ZNF350 (-29)	forward	AAAAACTCGAGTAAACCCGTGCGAGGACTCCAGAAG
ZNF350 (-13)	forward	AAAAACTCGAGCTCCAGAAGTAGGAGCAGTTTACGGAAG
ZNF350 (+49)	reverse	AAAAAAAGCTTTCTCCAGATACACAAGAAGGGCCTC
Primers for *ZNF350* coding sequence cloning		
ZNF350 (ENST00000243644)	forward	AAAAAGGATCCATGATCCAGGCCCAGGAATC
	reverse	AAAAAGCGGCCGCCTATGGGTTTTCTGTAACATA
Sequence of siRNAs		
Name		Sequence (5′-3′)
ZNF350 siRNA #1		GAAAUCAGGUCUCAUUAAA
ZNF350 siRNA #2		ACAGGAACGUAGUCCUUGU

Supplementary Table 2: Primers used for pyrosequencing experiment

Region	Forward primer (5'-3')	Reverse primer (5'-3')	Sequencing primer (5'-3')
1	GGAGTTAGGGAAG AAGAGAAGTT	Biotin-AACAATTTAACTT ACCCCATATTTACC	GGAAGAAGAGAAGTTATTG
2	Biotin-ATTTAAAATGTTTA AAAGAGTAAGGATAAG	TAACTTCTCTTCT TCCCTAACTCC	CTATACCTCCAATTTTCAAACATAA
3	GGTTTTTGGTTTAA AAATTTGTTATTGT	Biotin-AAACCACACACTA ACCTCTATTT	TTGTTTTTTTAAATATTTTAGGTTT
4	AGGATTTTAGAAG TAGGAGTAGT	Biotin-ACCACACAC TAACCTCTATT	ATTTTAGAAGTAGGAGTAGTTT

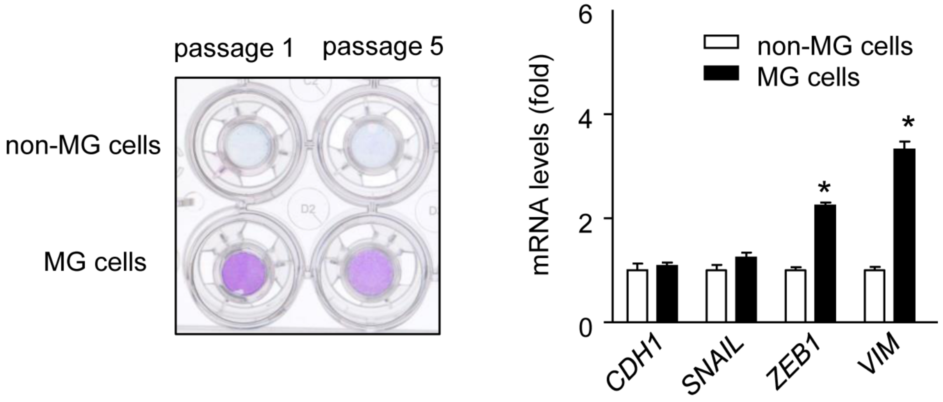

Supplementary Figure 1: Visualization of HCT116 cell migration for isolate MG and non-MG cells after one- or five passages under standard cell culture conditions. The migrating cells were visualized by Diff-Quick staining (left panel). Expression of *CDH1*, *SNAIL1*, *ZEB1,* and *VIM* mRNA in the both types of cells were assayed by qPCR using *GAPDH* mRNA as an endogenous quantitative control. Data are expressed as the mean relative changes ± SD (n = 4) compared to those in control non-MG cells. $^*P < 0.05$, unpaired Student's *t*-test.

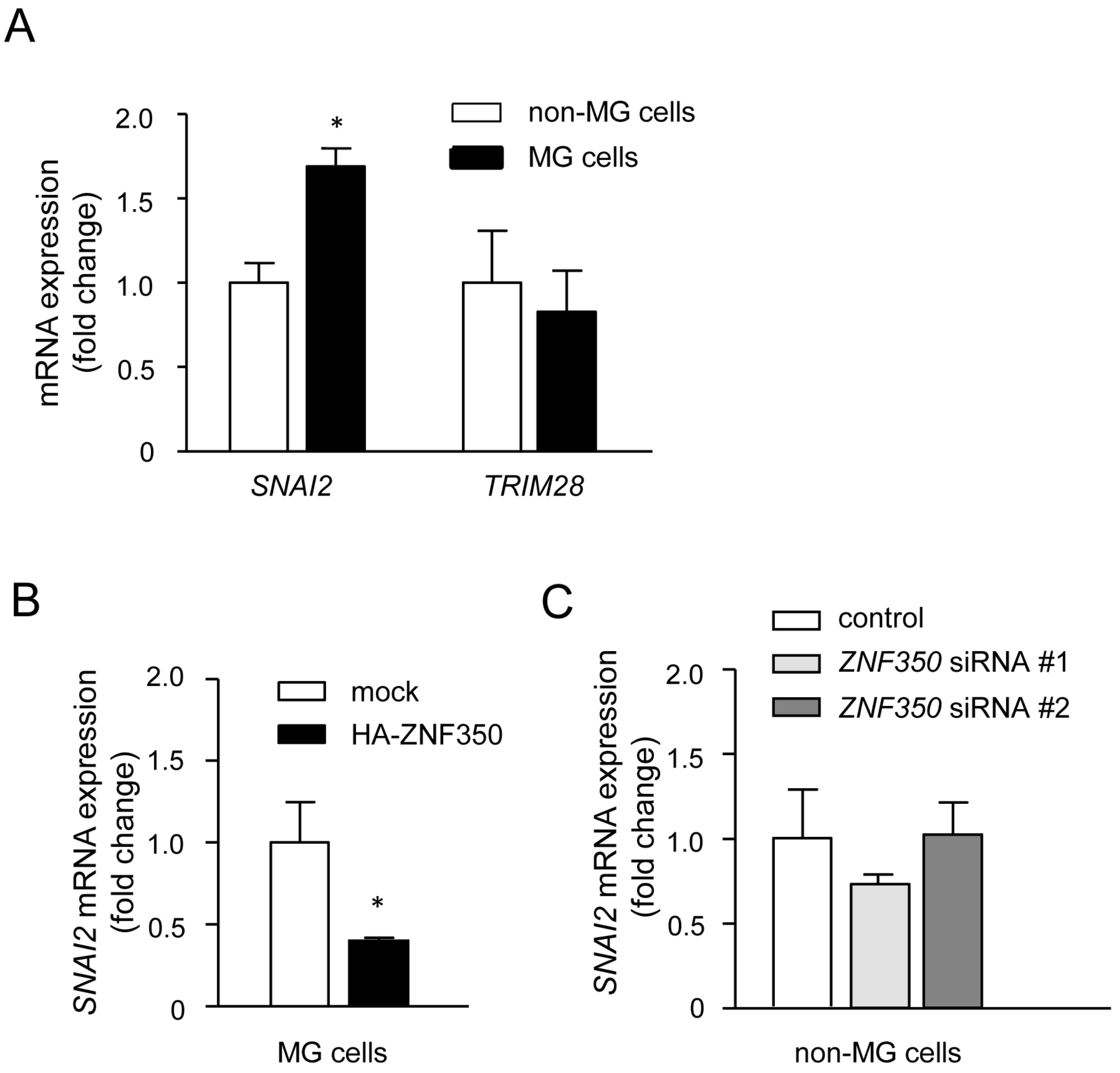

Supplementary Figure 2: (A) *SNAI2* and *TRIM28* mRNA levels in the MG and non-MG cells were assayed by qPCR. mRNA expression in the MG cells was calculated with the comparative ΔΔCt method using *GAPDH* mRNA as an endogenous quantitative control and are expressed as relative changes compared with their expression in the control non-MG cells. Data are presented as the means ± SD (n = 4). $^{*}P<0.05$, unpaired Student's *t*-test. **(B and C)** *SNAI2* mRNA levels in the MG cells transfected with mock or ZNF350 vector and in the non-MG cells transfected with siRNAs targeting *ZNF350* were assayed by qPCR. mRNA expression in the MG cells was calculated with the comparative ΔΔCt method using mock or control siRNA as an endogenous quantitative control and are expressed as the relative changes compared with expression in the control MG cells and non-MC cells. Data are presented as the means ± SD (n = 4). $^{*}P<0.05$, unpaired Student's *t*-test.

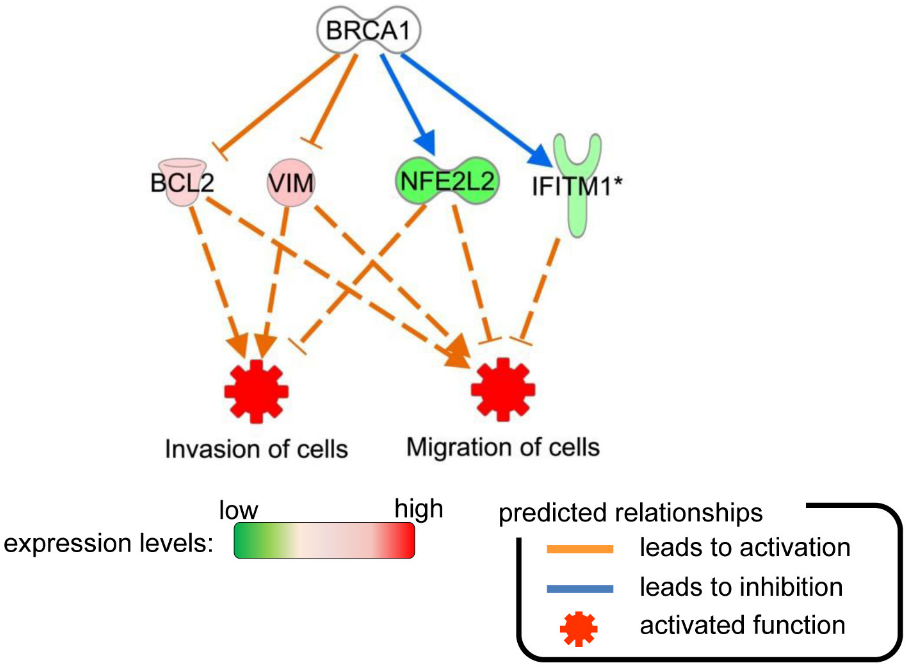

Supplementary Figure 3: Ingenuity pathway analysis (IPA) of differentially expressed *BRCA1*-related genes in the MG cells, focusing on the functions of migration and invasion of cells. Up-regulated and down-regulated genes in the MG cells are shown in red and green, respectively.
